# Health System Development in Nepal

**DOI:** 10.31729/jnma.4839

**Published:** 2020-01-31

**Authors:** Baburam Marasini

**Affiliations:** 1 Ex-Director, Epidemiology and Disease Control Division, Department of Health Services, Kathmandu, Nepal

**Keywords:** *Bir Hospital*, *Community health*, *Health*, *Nepal*

## Abstract

The development of health system in Nepal dates back from ancient period to the modern period starting from ayurvedic medicine to today's modern allopathic treatment. With regard to the different rulers in different time period, the number of improvements and changes in the health system of Nepal has been made. Moreover, the health system is further strengthened by the involvement of people and better management of health information and drug supply.

## INTRODUCTION

Nepal is continuously putting its efforts to develop a very competent health system in Nepal for the last 1500 years or so. Nepal was ruled by many dynasties and rulers during which they contributed well to develop the health system as far as the available resources allowed. In last 1500 years, three dynasties ruled in Nepal, and historians divided this period into the ancient period (ruled by Lichchhavi kings), the medieval period (ruled by Malla Kings and the modern period (ruled by Shah Kings).^1^

### EVOLUTION OF HEALTH SYSTEM IN NEPAL ANCIENT PERIOD (1-879 AD/56-935 BS)

In Ancient Nepal which accounts from 1^st^ to879 AD, the health system was based on traditional medicine that includes Ayurveda medicine in a major portion. During the Lichchhavi period, Aarogyashalawas established in Kathmandu valley and other parts of the country and these Aarogyashalawere probably Ayurveda medicine based health facilities. Ayurveda medicine started to grow during this period. Another public health policy intervention during this period was related to safe motherhood and executive order issued by the rulerAmshuVermawho started the system to cut the umbilical cord immediately after the baby was born, as the initial practice during that period was to cut the umbilical cord after the placenta is delivered.^1,2^

### MEDIEVAL PERIOD (880-1765 AD/936-182 BS)

During the Medieval period, Malla king JayasthitiMalla introduced state-accredited traditional birth attendants and prepared code of conduct and it says that they should provide service to everybody without discriminating caste and ethnicity. He also created a separate group for cutting the umbilical cord and disposal of it and this shows that the order issued by Lichchhavi RulerAmshuVerma was not fully complied. JayasthitiMalla also issued code of conduct for Baidhyas andAyuvedic practitioners. With the policy actions of Malla kings, the Ayurveda medicine got established as a family business during this period.^3^

Malla King PratapMallaestablished a state-funded Ayurveda dispensary in Hanuman Dhoka Royal Palace complex, probably to protect people from high costs of Ayurveda drugs and provide easy access to treatment.^4^ Malla Kings also introduced allopathic medicine in Nepal and they established three clinics in Kathmandu, Lalitpur and Bhaktapur respectively and allowed Christian missionary doctors to treat patients with allopathic or modern medicine. They treated smallpox patients and worked hard to eliminate the plague as a public health problem from Kathmandu. However, these clinics established by Christian missions closed in the year 1768 AD after the defeat of Malla Kings by the King of Gorkha Kingdom,Prithvi Narayan Shah.^5^

### MODERN PERIOD (RANA REGIME) (1766-1951 AD/ 1822-2007 BS)

In the modern period, Ayurveda medicine remained the mainstream health care system, as the Christian missionary clinics closed.In the year 1847 AD, Jung Bahadur Rana became the Prime minister of Nepal AD and initiated the Rana rule which continued till 1951. The Rana family rule continued for 104 years in Nepal and significant work was carried to developthe health system during that period.

In the era of 1850s, the major health problems were smallpox, cholera, malaria, leprosy and postpartum complications which contributed to significant morbidity and mortality. Rana Prime Minister Jung Bahadur initiated policy actions to strengthen the health system in Nepal. In the year 1850 AD, smallpox vaccination was started as a first health intervention which was based on modern medicine.^6^ In the same year, Baidhyakhana,as a dispensary, was established in Thapathali Durbar complex and this dispensary used to provide health services in both Ayurveda and allopathic medicine. Dr HA Oldfield,who worked as a medical doctor at British Residency, Lainchaur, Kathmandu during that period, was the first medical doctor to provide service and get remunerations from Government of Nepal.^7^ High ranking officials used to receive health services from ThapathaliBaidhyakhana by paying service charges and ordinary people used to receive from a separate clinic probably near Ranipokhari and it was a free clinic.^8,9^ Khokana Leprosy Asylum was the first health institution established by the state in 1857 AD to isolate the leprosy patients.^10^

Rana Prime Minister BirShumsher launched modern health care system as he established 15 bedded PrithviBir Hospital in Kathmandu in the year 1889 AD along with PrithviBir dispensaries in the same year in Hanuman Nagar, Jaleshwar, Birgunj, Taulihawa and Nepalgunj under KingPrithviBir Bikram Shah Dev.^11–13^ A separate leprosy clinic was also established in the same year in Teku, Kathmandu to treat leprosy patients.^12^ Rana prime minister further strengthened the health system by establishing several hospitals and dispensaries.

Rana Prime Minister Chandra Shumsher also continued to strengthen the health system by establishing hospitals and dispensaries, and in 1951 AD, all 35 districts had at least one hospital or dispensary. In this period, infectious disease hospitals such as Cholera hospital in Teku, Tuberculosis Sanatorium in Tokha, MalungaLeprosy Sanatorium in Syangja was established. Bir Hospital was also divided into Bir male and Bir female hospital to strengthen maternity services. During the Rana period, Ayurveda clinics were established throughout the country so as to provide access to alternative medicine. As a result, homoeopathic hospital and Unani dispensary were established.^14^ Under the Department of Health Services, Malaria Control Unit/ section were established and started functioning from ChisapaniGadhi, Makwanpur.^15^ It shows that the efforts of the Rana period to establish a national health system were praiseworthy.Health system was funded by land taxes during Rana period as land Birta and Guthi.^13^ However nowadays, Health system mainly funded by government, donors and INGOs.^16^

### Post-Democracy (After 2007 BS)

After the establishment of democracy in Nepal, the health services continued to expand along with the strengthening of the health system. In 1934 AD, Civil Medical School was established in Kathmandu with an aim to produce compounders and dressers.^8,9^ In 1954 AD, mission hospital was established in Tansen under United Mission to Nepal (UMN). UMN also established women's and children's welfare clinics in the Kathmandu Valley. In 1956 AD, Nepal Malaria Eradication Project was launched as a vertical project in 1958 AD. Several vertical projects were established to control and prevent Smallpox, Tuberculosis, Leprosy, Nutritional disorders, and Family Planning and maternal and child health issues. Health Assistant School and Nurse School was established in Kathmandu with the aim to produce paramedics and nurses. In 1956 AD, the government declared to establish one health centre in all 109 electoral constituencies and this scheme initiated health services at the sub-district level and also made policy decision to establish hospitals in all 35 districts. In 1961 AD, the government declared to establish zonal hospitals in all 14 zones to provide secondary health care under the new administrative reform.^17^ RajkiyaAyur vedicBidyalaya was established in 1972 BS at Nardevi, Kathmandu.

Until 1972 there were no medical schools in Nepal, with aspiring doctors having to pursue their studies in India and beyond. In the year 1972, the Institute of Medicine (IoM) under the Tribhuvan University, heralded a new dawn for medical education, allowing students to train as health professionals at home under the initiation of Dr. Moin Shah. Dr Shah upon his appointment as the dean immediately initiated courses for Auxiliary Nurse Midwives and Community Medical Assistants. Six years later, in 1978, he admitted 22 students as the first batch of MBBS students at the IoM. It then produced all categories of health workers and professionals. Initially Dr. Shah was criticized for being impractical, non-traditional and shunned by the world at large. What has happened instead is that the concepts that were then advocated —community medicine, problem solving, systematic instruction and integrated teaching—have now been widely accepted the world over. Institute of Medicine (IoM) started certificate level of programmes in Nursing, General Medicine, Health Laboratory, Pharmacy, Radiotherapy, Physiotherapy, Health Education and Sanitation.With the production of health workers by IoM, Government of Nepal declared to establish 1462 health posts in phases throughout the country to deliver basic health services in the year 1975 AD.^18^ Health for All strategy under the primary health care system adopted by the Government of Nepal in 1978. In 1978, Health training center was started to provide in-service training to health workers. The government initiated actions to create an integrated health system so as to phase out the then vertical project health programme in 1980.In 1986, the first regional hospital was established in Pokhara and continued to establish in other development regions as well. In 1956 AD In the same time-period, non-government hospitals were established to provide eye services in different parts of the country. In 1988, Mother's group and Female Community Health Volunteer Programme was initiated.^17^

In 1991, the first National Health Policy approved and started with the aim to establish one modern health care health facility (Primary health centres and sub-health posts) in all 4000 municipalities or village development committees. Few specialized hospitals were established to treat Non-communicable diseases.^19^ In 1991, the Government of Nepal adopted liberal economic policy and this opened the door for private medical colleges and private hospitals and they are now key players in the health system in Nepal.^20^ In 1993, National health training center was established along with five regional health training centers.^17^ In 2015, all sub-health posts upgraded to health posts.^21^ In 1968 Division of Indent, procurement and supply was established under Department of Health Services and it was later changed to Logistic Management Division in 1993. In 1993, the de-concentration of health management started and some management authorities delegated to municipalities and village development committees.^19^ The government established district health offices in all 75 districts and 5 regional health directorates.^22^ Integrated health management information system (HMIS) was initiated in 1994 under policy, Planning and Monitoring Division of Department of Health services.^23^

In 1996, the system for making hospital as an autonomous body by legislation was started and the first hospital, BP Memorial Cancer Hospital was established.^24^ Some devolution activities started in 2004 which was initially limited in some pilot districts.^25^ In 2017, the comprehensive devolution of basic health services to municipalities was done.^26^ Nepal Government Hospital and Dispensary Office was later upgraded to Department of Health Services in 1934.^27^ The involvement of community could be seen in health system of Nepal as female community health volunteer and mother's groups. Moreover, people have started to donate land to construct health facilities and are also providing monetary contribution.^18^

### WAY FORWARD

Nepal has its' own system of indigenous system of medicine which remained mainstream health system till initial part of modern Nepal. There was inter-relation of health and religion in ancient days.The traditional medicine could not develop as an effectivehealth care system despite of the main health system of thecountry for more than one century.History of modern health services is not long in Nepal thoughthe Christian missionaries were introduced it in 16th century.There seems lack of better management of health institution and lack of effective health policy.

## Conflict of Interest

**None.**
